# Vacuolar Phosphate Transporter1 (VPT1) may transport sugar in response to soluble sugar status of grape fruits

**DOI:** 10.1093/hr/uhac260

**Published:** 2022-11-22

**Authors:** Qian Bai, Xuexue Chen, Zhenzhen Zheng, Jinjing Feng, Yanjun Zhang, Yuanyue Shen, Yun Huang

**Affiliations:** Beijing Key Laboratory for Agricultural Application and New Technique, College of Plant Science and Technology, Beijing University of Agriculture, Beijing 102206, China; Ministry of Education Key Laboratory of Silviculture and Conservation, College of Forestry, Beijing Forestry University, 35 East Qinghua Road, Beijing 100083, China; Beijing Key Laboratory for Agricultural Application and New Technique, College of Plant Science and Technology, Beijing University of Agriculture, Beijing 102206, China; Beijing Key Laboratory for Agricultural Application and New Technique, College of Plant Science and Technology, Beijing University of Agriculture, Beijing 102206, China; Beijing Key Laboratory for Agricultural Application and New Technique, College of Plant Science and Technology, Beijing University of Agriculture, Beijing 102206, China; Beijing Key Laboratory for Agricultural Application and New Technique, College of Plant Science and Technology, Beijing University of Agriculture, Beijing 102206, China; Beijing Key Laboratory for Agricultural Application and New Technique, College of Plant Science and Technology, Beijing University of Agriculture, Beijing 102206, China; Beijing Key Laboratory for Agricultural Application and New Technique, College of Plant Science and Technology, Beijing University of Agriculture, Beijing 102206, China

## Abstract

Vacuolar Phosphate Transporter1 (VPT1)-mediated phosphate uptake in the vacuoles is essential to plant development and fruit ripening. Interestingly, here we find that the VPT1 may transport sugar in response to soluble sugar status of fruits. The VvVPT1 protein isolated from grape (*Vitis vinifera*) berries was tonoplast-localized and contains SPX (Syg1/Pho81/XPR1) and MFS (major facilitator superfamily) domains. Its mRNA expression was significantly increased during fruit ripening and induced by sucrose. Functional analyses based on transient transgenic systems in grape berry showed that VvVPT1 positively regulated berry ripening and significantly affected hexose contents, fruit firmness, and ripening-related gene expression. The VPT1 proteins (Grape VvVPT1, strawberry FaVPT1, and Arabidopsis AtVPT1) all showed low affinity for phosphate verified in yeast system, while they appear different in sugar transport capacity, consistent with fruit sugar status. Thus, our findings reveal a role for VPT1 in fruit ripening, associated to its SPX and MFS domains in direct transport of soluble sugar available into the vacuole, and open potential avenues for genetic improvement in fleshy fruit.

## Introduction

The mineral macroelement phosphorus (P) is essential for plant growth and inorganic phosphate (Pi) is a major available transportable form of P from soil to cells. To understand the regulation mechanism of Pi homeostasis in plants, previous studies have identified the transporters involved in Pi uptake, translocation, and storage [1]. For example, the Pi influx transporters *Arabidopsis thaliana* Vacuolar Phosphate Transporter1 (AtVPT1/AtPHT5;1), strawberry (*Fragaria* × *ananassa*) FaVPT1, and the *Oryza sativa* vacuolar Pi efflux transporters OsVPE1 and OsVPE2 contribute to the maintenance of Pi homeostasis in the vacuole [2–5]. The AtVPT1, FaVPT1, and OsVPE1/2 proteins, which belong to the major facilitator superfamily (MFS), are localized to the tonoplast and affect vacuolar Pi content and plant adaptation to Pi status, which is crucial for crop yield and fruit quality.

Sugar and phosphate may interact with each other during organism growth and development [6]. Cytosol-vacuole Pi flux largely depends on proton gradient across tonoplast, which is tightly associated with carbohydrate status and sugar transport. Sugar transport has essential functions in the allocation of photosynthate to sink organs for growth and energy storage [7]. Furthermore, many fleshy fruits store sugars and P in their vacuoles [8]. Notably, the first report in fleshy fruit finds that strawberry FaVPT1 transports Pi into vacuoles and promotes fruit ripening by sugar signaling [2], which determines fruit quality. However, the defined mechanism remains unknown.

Given that MFS proteins can transport numerous transmembrane-trafficking substrates [9], and in particular the main sugar family transporters, including monosaccharide transporters (MSTs) and sucrose transporters (SUTs) belong to the MFS [10], this provokes us to explore the direct relationship of VPT1 proteins with sugar. AtPHT4;4, a member of the phosphate transporter 4 family of *A. thaliana*, functions as an ascorbate transporter at the chloroplast envelope membrane, associated to the adaptation to strong light stress [11]. The Nitrate Transporter 1/Peptide Transporter (NRT1/PTR) ZmSUGCAR1/ZmNPF7.9 belonged to MFS encodes a low-affinity nitrate transporter, which is capable of transporting sucrose and glucose as substrates [12]. Thus, plant MFS members have been shown to transport a wider variety of substrates.

In the present study, using a variety of approaches, we identified the VvVPT1 from grape (*Vitis vinifera*), a tonoplast located transporter, as a key factor in the regulation of berry hexose accumulation and ripening, also strikingly finding that VvVPT1 could transport sugar. In comparison to the VPT1 available (only strawberry FaVPT1 and Arabidopsis AtVPT1), as expected, the three proteins, VvVPT1, FaVPT1, and AtVPT1, all have low-affinity Pi transport activity. Interestingly, the three proteins have different sugar transport capacity: (i) VvVPT1 has high-affinity for both glucose and fructose; (ii) FaVPT1 has high-affinity for sucrose; while (iii) AtVPT1 has no sugar transport capacity, consistent with their sugar status that strawberry fruit mainly accumulate sucrose, the grape berries main accumulate glucose and fructose, the Arabidopsis fruit belongs to dried fruit with no soluble sugar. To our knowledge, this is the first report on the specificity of MFS proteins associated with sugar and phosphate trafficking in vacuoles, providing an insight into P/sugar transport in economically important fruits.

## Results

### Spatial and temporal expression of VvVPT1

AtVPT1 (AT1G63010) is responsible for vacuolar Pi transport and essential for Pi adaptation in Arabidopsis [3]. In strawberry, FaVPT1 (XP_004298733.1) affects not only fruit phosphorus content but also sugar accumulation [2]. The function of VPT1 homologous protein in grape, which is a typically fleshy fruit rich in sugars, has not been characterized to date. The amino acid sequence of FaVPT1 was used for BLAST search in the NCBI databases to find the homologous protein in *V. vinifera*. The sequence from *V. vinifera* showed 93% and 88% similarity with FaVPT1 and AtVPT1 protein, thus the protein was named VvVPT1 (VIT_202s0025g04540; Fig. S1a, see online supplementary material). Transmembrane (http://www.sbg.bio.ic.ac.uk/phyre2) and conserved domain (https://www.ncbi.nlm.nih.gov/cdd) prediction showed that VvVPT1 belongs to the SPX-MFS family containing 12 transmembrane domains (Fig. S1b, see online supplementary material).

**Figure 1:**
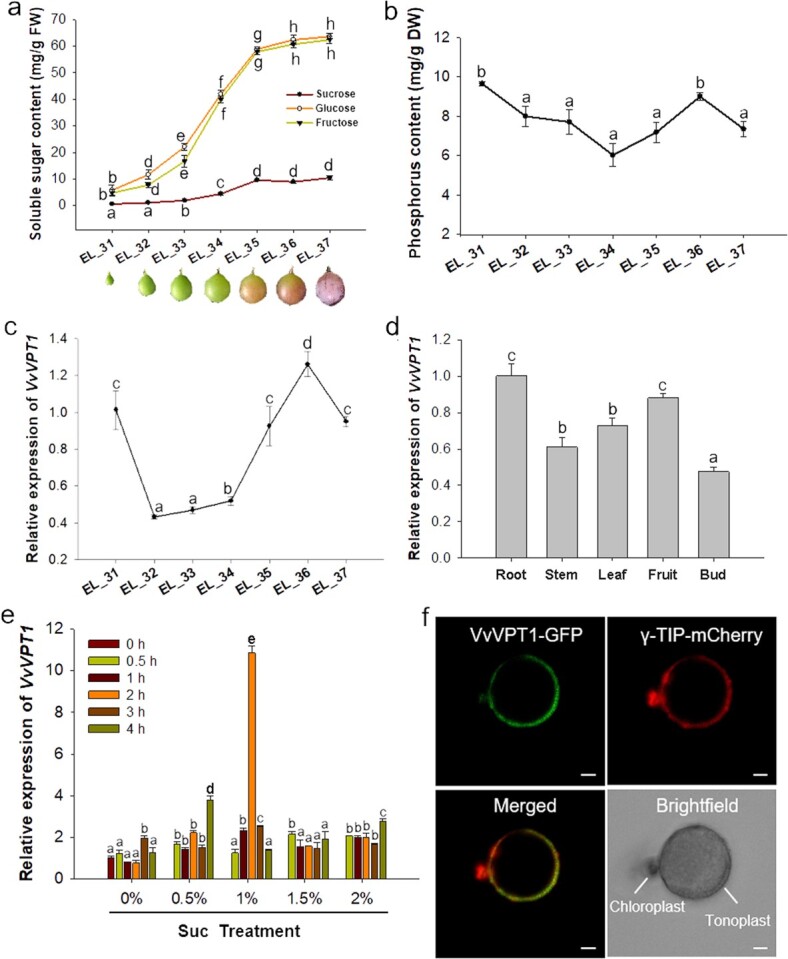
Expression pattern and subcellular localization of VvVPT1. **a** The content of sugar in berries at different developmental stages. Error bars represent SE (*n* = 3). Photos of seven development stages of *Vitis vinifera* ‘Kyoho’ fruit were shown. **b** Phosphorus in berries at different developmental stages. Error bars represent SE (*n* = 3). **c**−**d** The expression level of *VvVPT1* at different developmental stages of *V. vinifera* ‘Kyoho’ fruit and different tissue. The fruit is referred to EL_37 developmental stage. **e** qPCR analysis of *VvVPT1* expression in response to different concentration of Suc. Data are shown as the mean ± SE (*n* = 3). The flesh at EL_34 stage was cut into 1 mm discs, and then equally distributed to 250 mL flasks containing incubate liquid with different concentrations of Suc for 0.5 h, 1 h, 2 h, 3 h, and 4 h. The discs incubated for 30 min were used as control. **f** Subcellular localization of VvVPT1. The panels show the GFP signal (green), the mCherry signal (red), an overlay of the GFP and mCherry signals, and the bright field image from the same sample. γ-TIP is a tonoplast marker. Bars: 5 μm. Different letters (a, b, c, d, e, f, g, and h) above the columns indicate statistically significant difference (*P* < 0.05).

To investigate the function of VvVPT1 in grape, we firstly determined the expression patterns and subcellular localization of *VvVPT1*. The berry contents of sugar and phosphorus were determined during seven developmental stages including the expanding period (EL_31 to EL_34), véraison period (EL_35), and mature period (EL_36 to EL_37) of ‘Kyoho’ (*V. vinifera*) (Fig. 1a). Consistent with previous reports, ‘Kyoho’ fruits mainly accumulate hexose, almost no sucrose (Suc). The content of hexose including glucose (Glc) and fructose (Fru) accumulated rapidly, while Suc increased slowly during grape fruit development (Fig. 1a). Total phosphorus content in grape berry was also measured. It decreased from the EL_31 to EL_34, EL_36 to EL_37, but increased from EL_34 to EL_36 (Fig. 1b). In comparison to the sugar content, the phosphorus content in grape berry changed slightly during berry development and ripening.

Next, *VvVPT1* was found to be expressed at all stages of grape berry development analysed by qRT-PCR. The expression of *VvVPT1* strongly decreased after fruit setting, but increased remarkably from EL_32 to EL_36 and reached the peak about 10 d after véraison (Fig. 1c). *VvVPT1* was expressed throughout the plant, including the roots, stems, leaves, fruits (matured), and buds (Fig. 1d). The impact of Suc, Glc, and Fru on the expression level of *VvVPT1* was tracked through the fruit disc incubation *in vitro*. The results of qRT-PCR analysis showed that 1% Suc treated for 2 h significantly promoted *VvVPT1* expression compared to the Glc and Fru treatment (Fig. 1e; Fig. S2, see online supplementary material). Interestingly, nine Suc-responsive element (SURE) like sequences (seven AATAAAA, one AATAAATAAA, and one AAAATCA—TAA; Fig. S3, see online supplementary material), four G-box, and two W-box elements were identified in the 1500 bp upstream of the ATG initiation codon of *VvVPT1*. These *cis* elements have been widely identified in sugar-regulated plant promoters [13, 14].

To determine the subcellular localization of VvVPT1, we transiently expressed the VvVPT1 protein fused with green fluorescence protein (GFP) in *Nicotiana benthamiana* leaves. The GFP fluorescence was visible on the vacuolar membrane, when the central vacuole was released from protoplasts using osmotic shock treatment. VvVPT1-GFP signal overlapped with the tonoplast marker, the γ-TIP-mCherry fusion protein (Fig. 1f). These results revealed that VvVPT1 was localized to the vacuolar membrane.

### VvVPT1 is required for hexose accumulation in grape berry

**Figure 2:**
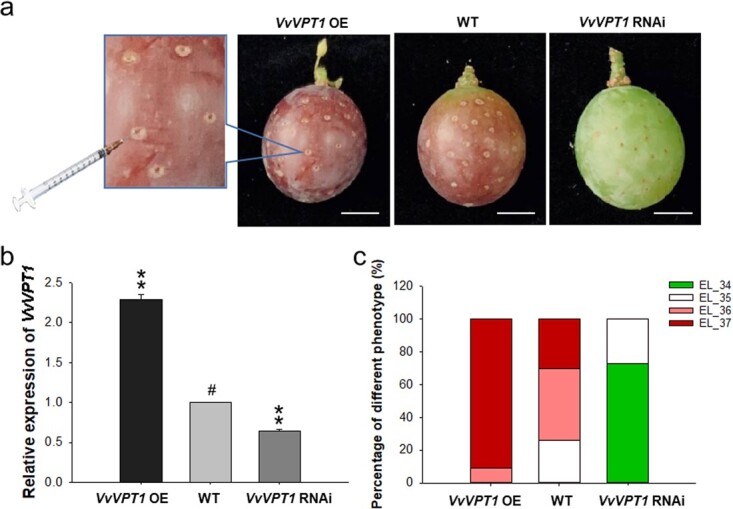
Phenotypes of *VvVPT1* RNAi and *VvVPT1* OE transgenic fruits. **a**  *Agrobacterium* GV3101 strains containing *VvVPT1* RNAi (RNA interference) or *VvVPT1* OE (overexpression) recombinant plasmids were injected into grape berries. The fruit phenotype was photoed at 7 DAI. Bars: 1 cm. **b** Analysis of *VvVPT1* transcript levels in RNAi and OE fruits compared with the control. *VvActin* mRNA was used as an internal control. The asterisks (^**^) above the columns indicate statistically significant differences (*P* < 0.01) compared with the control. **c** Phenotypic statistics of *VvVPT1* RNAi and *VvVPT1* OE *Vitis vinifera* ‘Kyoho’ fruit. The ordinate represents the proportion of transgenic fruits at different development stages. Thirty berries were used for each treatment and the experiment was repeated at least three times.

According to the previous results, we predicted that the VvVPT1 may be of importance in grape berry ripening. Considering the inefficient transformation and the relatively long growth cycle of grape, we carried out functional analyses of VvVPT1 using a newly designed transgenic system in grape berry [15]. *Agrobacterium* GV3101 strains transformed *VvVPT1* RNAi (intron-hairpin RNA interference) or *VvVPT1* OE (overexpression) were injected into fruits attached to *V. vinifera*. Phenotypes were assessed at 7 d after injection. Colour development of the grape peel was markedly inhibited in the *VvVPT1* RNAi fruits, while accelerated in the *VvVPT1* OE fruits (Fig. 2a). Expression level of *VvVPT1* in the transgenic fruits was confirmed by qRT-PCR analyses, significantly downregulated and upregulated in the RNAi and OE fruits compared with the control (Fig. 2b). According to the phenotype analysis of transgenic fruits, it was found that more than 85% of *VvVPT1* OE fruits were at EL_37 stage with purple peel, while more than 75% of *VvVPT1* RNAi fruits were at EL_34 stage with green peel (Fig. 2c). These data indicated that VvVPT1 positively contributed to regulate grape berry ripening.

**Figure 3:**
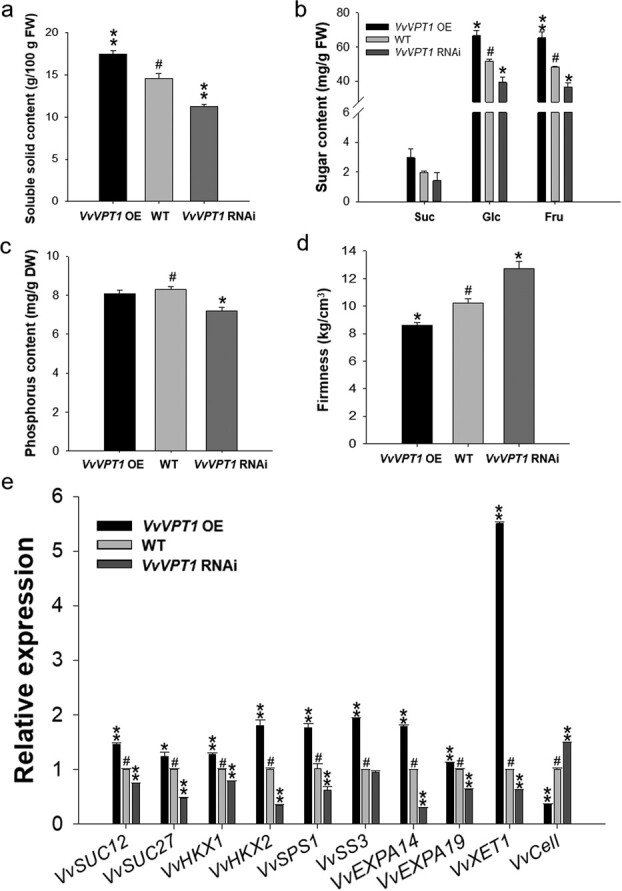
Physiological parameters and transcript levels of ripening-related genes in *VvVPT1* transgenic fruits. **a** Soluble solids concentration in *VvVPT1* transgenic fruits. **b** Sugar content (Suc, Glc, and Fru) in *VvVPT1* transgenic fruits. **c** Total phosphorus content in *VvVPT1* transgenic fruits. **d** Fruit firmness in *VvVPT1* transgenic fruits. **e** Transcript levels of ripening-related genes detected by qRT-PCR. *VvActin* mRNA was used as an internal control. Data are shown as the mean ± SE (*n* = 3). Asterisks indicate significant differences compared with the control fruit: ^*^P < 0.05; ^**^P < 0.01. OE: overexpression; RNAi: RNA interference; *VvSUT12*: Suc transporter12; *VvSUT27*: Suc transporter27; *VvHXK1*: hexokinase1; *VvHXK2*: hexokinase2; *VvSPS1*: Suc phosphate synthase1; *VvSS3*: Suc synthase3; *VvEXPA14*: expansina14; *VvEXPA19*: expansina19; *VvXET1*: Xyloglucan endotransglycosylase1; *VvCEll*: cellulose.

To further investigate the function of VvVPT1, we analysed important physiological changes of the transgenic RNAi and OE grape berry, including soluble solids concentration, sugar content (Glc, Fru, and Suc), total phosphorus content, and fruit firmness. The soluble solids concentration, Glc, and Fru contents were significantly increased in OE fruits and decreased in RNAi fruits compared with the control fruits (Fig. 3a and b), while there was no significant difference in Suc content in the transgenic fruits compared with the control fruits (Fig. 3b). Total phosphorus content of RNAi fruits was significantly lower than the control, while there was no significant difference between OE fruits and the control fruits (Fig. 3c). Fruit firmness significantly declined in OE fruits and increased in RNAi fruits compared with the control fruits (Fig. 3d).

In order to prove that VvVPT1 regulated sugar accumulation and fruit firmness in grape at molecular level, we also measured the transcription of genes associated with fruit sugar accumulation such as Suc transporter12 gene (*VvSUT12*, VIT_201s0026g01960), Suc transporter27 gene (*VvSUT27*, VIT_218s0076g00250), hexokinase1 gene (*VvHXK1*, VIT_209s0002g03390), hexokinase2 gene (*VvHXK2*, VIT_218s0001g14230), Suc phosphate synthase1 gene (*VvSPS1*, VIT_204s0008g05730), and Suc synthase3 gene (*VvSS3*, VIT_207s0005g00750), and the softening-related genes such as expansion genes (*VvEXPA14*, VIT_13s0067g02930; *VvEXPA19*, VIT_18s0001g01130), xyloglucan endotransglycosylase1 (*VvXET1*, VIT_201s0150g00460), and endoglucanase (*VvCell*, VIT_204s0044g00780). qRT-PCR analysis showed that *VvSUT12*, *VvSUT27*, *VvHXK1*, *VvHXK2*, *VvSPS1*, *VvSS3*, *VvEXPA14*, *VvEXPA19*, and *VvXET1* were downregulated and upregulated in the RNAi and OE fruits, respectively, compared with the control fruits (Fig. 3e). Only expression of *VvCell* upregulated and downregulated in the RNAi and OE fruits (Fig. 3e). The changes in the transcription of genes associated with sugar and firmness in the transgenic fruit indicated that VvVPT1 positively regulated the fruit ripening and quality.

### VvVPT1, FaVPT1, and AtVPT1 show conserved Pi transport ability but distinct sugar-uptake functions in yeast cells

According to the putative three-dimensional structures, the structures of FaVPT1, VvVPT1, and AtVPT1 proteins are similar to the sugar transporter structure MFS with 100% confidence (Fig. S4, see online supplementary material; PDB number:3O7P, 6KKL, 6E9O). Moreover, FaVPT1 and VvVPT1 promoted fruit ripening and sugar accumulation. Therefore, FaVPT1, VvVPT1, and AtVPT1-mediated transport of Pi and sugar were investigated through three mutant strains in the yeast system as speculated that the function of VPT1 proteins is likely associated with sugar transport. We heterologously expressed VPT1 proteins in yeast (*Saccharomyces cerevisiae*) and demonstrated that they can express in the yeast system by the subcellular localization experiments (Fig. S5, see online supplementary material).

**Figure 4:**
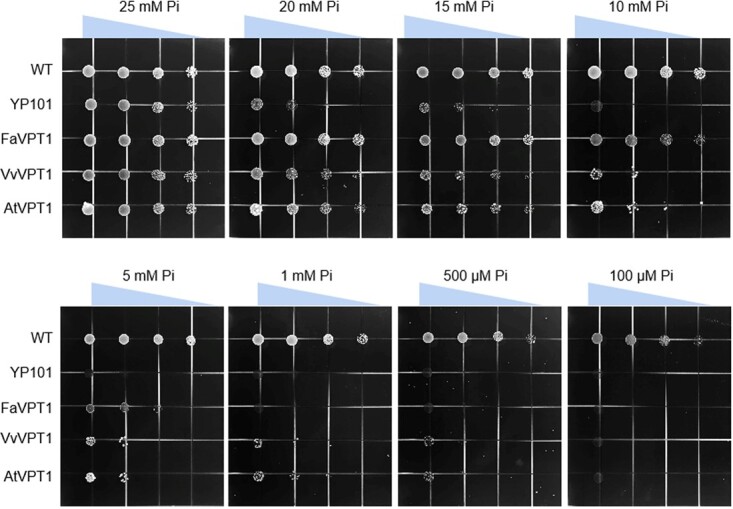
Inorganic phosphate (Pi) uptake activity analysis in yeast system. The yeast mutant strain YP101, which has defective Pi uptake, was transformed with *FaVPT1*, *VvVPT1*, and *AtVPT1*, respectively. Yeast grown on medium fortified with different concentration of Pi. WT: wild type yeast. Strains were diluted to OD_600_ values of 2, 0.2, 0.02, and 0.002 on the plates. All plates were incubated at 30°C for 5 d. The triangles on the top of the graphs mean different yeast concentrations.

Firstly, to evaluate the Pi transport abilities of VPT1 proteins, the complementation assay was performed using the Pi uptake deficient yeast strain YP101 (*pho84*Δ *pho87*Δ *pho89*Δ *pho90*Δ *pho91*Δ) [16]. The wild type (WT) yeast grew well on the plates supplied with different levels of Pi, and YP101 could grow on 25 mM and 20 mM Pi supplied medium (Fig. 4). The yeast mutant transformed with *FaVPT1*, *VvVPT1*, or *AtVPT1* restored growth and showed a more vigorous growth over YP101 from 20 mM Pi to 1 mM Pi (Fig. 4). These data indicated that VvVPT1 showed Pi transport activity in a heterologous system similar to FaVPT1 and AtVPT1, and all serve as low-affinity Pi transporters, confirming a conserved function of the three proteins in phosphate transport.

**Figure 5:**
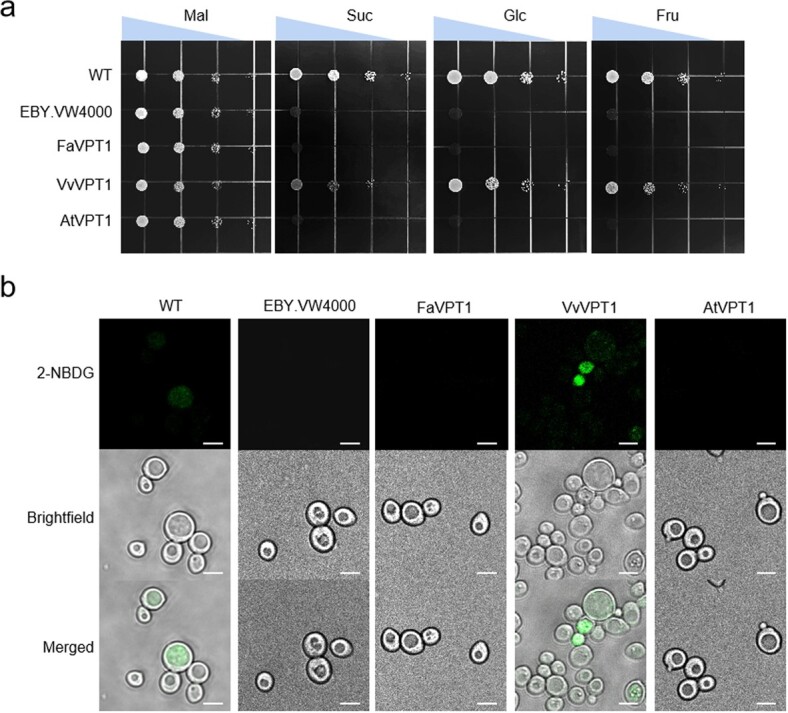
Growth and 2-NBDG uptake of EBY.VW4000 yeast (*Saccharomyces cerevisiae*) strain. **a** Growth of WT, EBY.VW4000, and EBY.VW4000 transformed with *FaVPT1*, *VvVPT1*, or *AtVPT1* on 2% maltose (Mal), sucrose (Suc), glucose (Glc), or fructose (Fru). The triangles on the top of the graphs mean different yeast concentrations. **b** Uptake of the fluorescent derivative of D-Glc, 2-NBDG, in yeast strain EBY.VW4000 expressing *FaVPT1*, *VvVPT1*, or *AtVPT1*. Bars: 5 μm.

Secondly, to investigate if the three VPT1s functioned in hexose transport, we used the yeast strain EBY.VW4000, which is defective in hexose transport and cannot grow on the medium with hexose or Suc as a sole carbon source, but could grow on maltose (Mal) normally due to the intracellular maltase [17]. The *VvVPT1*-expressed yeast strain grew on 2% (w/v) Glc, Fru, and Suc medium normally while *FaVPT1* or *AtVPT1* could not (Fig. 5a).

To further confirm the capacity of VvVPT1 in Glc transport in yeast, we recruited a fluorescent derivative of D-Glc, 2-[N-(7-nitrobenz-2-oxa-1,3-diazol-4-yl) amino]-2-deoxy-d-Glc (2-NBDG), which is used for the analysis of Glc uptake activity in living cells [18, 19]. Microscopy analysis showed that WT yeast strain could take up 2-NBDG and appeared with green fluorescence. The fluorescence in *VvVPT1-*expressed yeast strain was stronger than that of the WT (Fig. 5b), while the fluorescence could not be detected in the EBY.VW4000 transformed with *FaVPT1* or *AtVPT1*. These results further supported that VvVPT1 possibly functioned as a glucose transporter.

**Figure 6:**
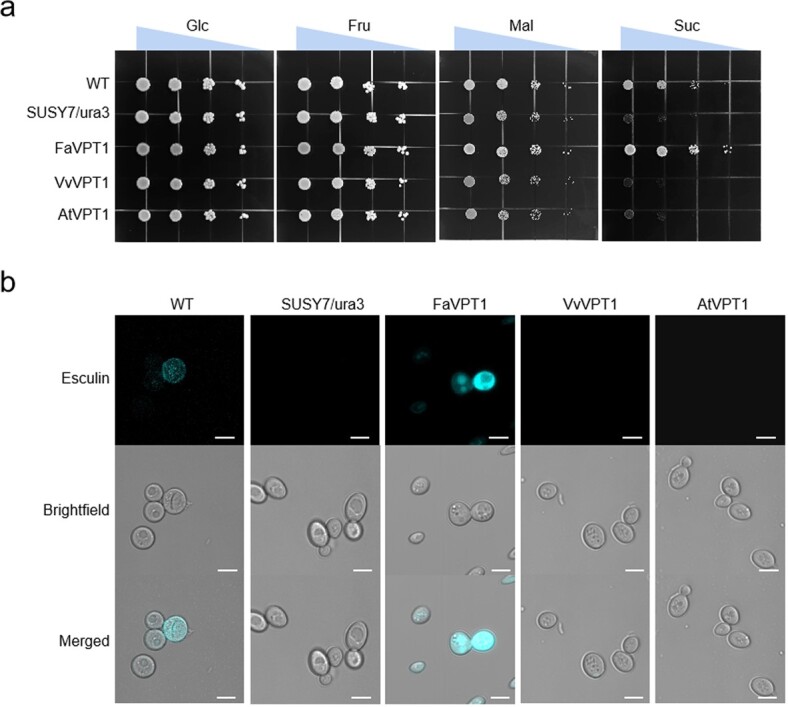
Growth and esculin uptake of Susy7/ura3 yeast (*Saccharomyces cerevisiae*) strain. **a** Growth of WT yeast strain, Susy7/ura3, and Susy7/ura3 transformed with *FaVPT1*, *VvVPT1*, or *AtVPT1* on 2% maltose (Mal), sucrose (Suc), glucose (Glc), or fructose (Fru). The triangles on the top of the graphs mean different yeast concentrations. **b** Uptake of the sucrose analogue esculin in yeast strain Susy7/ura3 expressing *FaVPT1*, *VvVPT1*, or *AtVPT1* and visualized by fluorescence microscopy. Bars: 5 μm.

In addition, *AtVPT1*, *FaVPT1*, and *VvVPT1* were integrated into the mutant yeast strain SUSY7/ura3 defective in sucrose transport, respectively [20, 21]. The results showed that the SUSY7/ura3 yeast strains carried with *FaVPT1*, *VvVPT1*, or *AtVPT1* are able to grow on the medium supply of Glc, Fru, or Mal. Only WT and the SUSY7/ura3 yeast strain expressed *FaVPT1* could grow on the medium with 2% (w/v) Suc as sole carbon source (Fig. 6a). Given this yeast mutant cannot survive using sucrose as sole carbon source [22], these results therefore suggested that among these transporters, only FaVPT1 has sucrose transport capacity.

**Figure 7:**
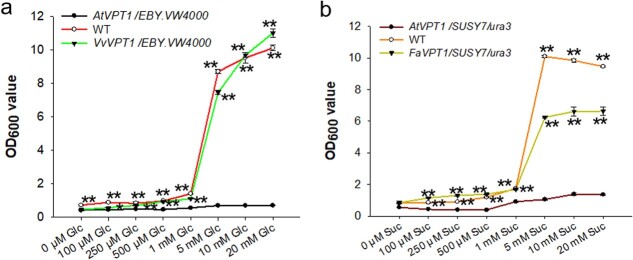
The growth curve of yeast cells under different concentrations of sugar. Growth curve of yeast cells under different concentrations of Glc (**a**) and Suc (**b**). Yeast growth was determined at 600 nm (OD_600_) after 48 h at 30°C. Data are shown as the mean ± SE (*n* = 3), ^*^P < 0.05; ^**^P < 0.01.

To further confirm the capacity of FaVPT1 in sucrose transport, a mimic uptake of Suc in yeasts was performed via esculin [23, 24]. Incubation of the different transformed yeast strains showed that both WT and the *FaVPT1*-expressed yeast cells had significantly fluorescence intensity, but no fluorescence appeared in the SUSY7/ura3 expressed *VvVPT1* or *AtVPT1* (Fig. 6b). The fact that the *FaVPT1*-expressed yeast strain could take up both esculin and sucrose demonstrated that FaVPT1 has sucrose uptake capacity while VvVPT1 and AtVPT1 does not.

To assess the uptake affinity of sugars with FaVPT1 and VvVPT1, the growth curves of yeast supplied with different concentration of sugars were recorded. The growth rate of mutant yeast strain (SUSY7/ura3 or EBY.VW4000) rised slowly with an increase in the concentrations of Suc or Glc (Fig. 7). The yeast strain transformed with *FaVPT1* or *VvVPT1* grew significantly faster than the mutant strain over a range of sugar concentrations (100 μM to 20 mM; Fig. 7). The growth rate of the *VvVPT1* transformed strain was similar to WT (Fig. 7a), but the *FaVPT1* transformed strain grew more slowly than WT (Fig. 7b). These results indicated that FaVPT1 and VvVPT1 have high affinity to sugars.

### The SPX domains of VvVPT1, FaVPT1, and AtVPT1 determine the transport ability of Pi and sugar in yeast cells

It is reported that the alternating-access mechanism of the MFS transporters by a switch between outward-facing and inward-facing conformation allow alternating access to the substrate binding site from either side of the membrane [10]. On the basis of the predicted three-dimensional structures of FaVPT1, VvVPT1, and AtVPT1 (Fig. S4, see online supplementary material) and the SPX domain acting as a sensor to control phosphate homeostasis [2, 25], we hypothesize that the SPX domain of VPT1s may affect its substrate binding.

**Figure 8:**
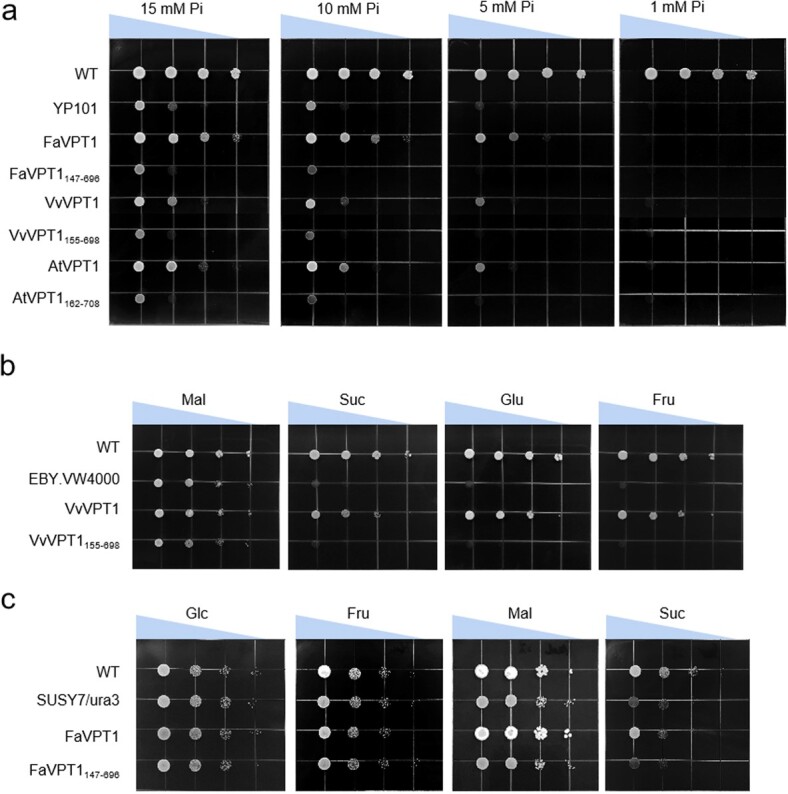
Deletions of the SPX domains affect VPT1 proteins’ transport capacity in yeast. **a** The yeast mutant strain YP101 was transformed with *FaVPT1*, *VvVPT1*, *AtVPT1*, and the VPT1s truncating the SPX domain, respectively. Yeast grown on medium fortified with different concentration of Pi. WT: wild type yeast. Strains were diluted to OD_600_ values of 2, 0.2, 0.02, and 0.002 on the plates. All plates were incubated at 30°C for 5 d. **b** Growth of WT, EBY.VW4000, and EBY.VW4000 transformed with *VvVPT1*, or *VvVPT1* with SPX domains deleted (VvVPT1_155–698_) on 2% maltose (Mal), sucrose (Suc), glucose (Glc), or fructose (Fru). **c** Growth of WT, Susy7/ura3, and Susy7/ura3 transformed with *FaVPT1* or *FaVPT1* with SPX domains deleted (FaVPT1_147–696_) on 2% maltose (Mal), sucrose (Suc), glucose (Glc), or fructose (Fru).

We constructed truncated versions of the VPT1 proteins to test whether the SPX domain is required for their transport ability. FaVPT1_147–696_, VvVPT1_155–698_, and AtVPT1_162–708_ were transformed into yeast mutant strains YP101, SUSY7/ura3, or EBY.VW4000, respectively. The results demonstrated that VPT1 proteins without the SPX domain could not transport Pi, sucrose, or hexose (Fig. 8a−c), confirming that the SPX domain is indispensable for the transport function of VPT1. Taken together, to a large degree, the integration presence of the SPX and MFS domains into the VPT1 protein is important for its function.

## Discussion

### Tonoplast located VvVPT1 plays an important role in hexose accumulation and grape berry ripening

Grape (*V. vinifera*) is used worldwide as fresh-eating and in wine making. The organoleptic quality of the berries and the flavor and stability of wine are determined by the type and concentration of sugars and acids in grapes [26]. Fruit soluble sugars (Suc, Glu, and Fru) from leaf photoassimilates mainly stored in vacuoles and determine sweetness, an important property of fruits [27, 28]. Phosphorus fertilizer is one of the indispensable nutritional elements for fruit growth and development. Phosphorus can promote flower bud differentiation, fruit ripening, and improve fruit quality. However, little attention was paid to the relationship between phosphorus and grape berry sugar accumulation. In this study, the SPX-MFS phosphate transporter VvVPT1 was identified and highly expressed at grape berry after véraison. We demonstrated that VvVPT1 functions in positively regulating hexose accumulation potentially by directly transporting hexose into the vacuole and thereby enhances berry ripening.

In general, inverse-concentration accumulation of soluble sugar in cells and vacuoles is mediated by sugar transporters, including MSTs, SUTs, and SWEETs, among which MSTs and SUTs belong to the MFS proteins with 12 transmembrane domains [10, 29]. In grape berries, hexose transporter VvHT1 and Tonoplast Monosaccharide Transporters TMT1 and TMT2 play a vital role in sugar accumulation [30, 31]. Sucrose transporters VvSUC11, VvSUC12, and VvSUC27 have been identified and their overexpressing tomatoes significantly improved the sugar content of tomato fruits [32]. In addition, whether these reported MFS superfamily sugar transporters is also related to phosphate transport is to be studied.

As we described above, a rise in the relative rate of sugar accumulation between EL_32 and EL_36 was correlated with the peak of *VvVPT1* expression, although expression of *VvVPT1* rapidly decreased after fruit setting and before harvest (Fig. 1c). These results showed that the accumulation of *VvVPT1* transcripts may be controlled precisely. Meanwhile, *VvVPT1* transcription was induced by sucrose (Fig. 1e), similar to that of *FaVPT1* [2]. This is because sucrose not only serves as a major photosynthate that is transported from leaves to fruits in plants such as strawberry and grape [33], but it also has a signaling function during fruit ripening [34, 35]. Although 1% Suc can promote *VvVPT1* expression compared to the Glc and Fru treatment (Fig. 1e; Fig. S2, see online supplementary material), the lesser content of Suc in the berry fruit limits its role, this is also consistent with no significant difference in Suc content in the transgenic fruits compared with the control fruits (Fig. 3b). Thus, these results suggest a dominant role of VvVPT1 in transport monosaccharide.

### VPT1 proteins mediated transport of sugars and phosphate differs among fruit types

In land plants, flowering plants have evolved both dry and fleshy fruits. In general, the maturation of dry fruits is coupled with dehydration and dehiscent; by contrast, the ripening of fleshy fruits is concomitant with vacuole expansion. Fleshy fruits like grape and strawberry are rich in water, sugars, and secondary metabolites in the vacuoles, which are beneficial to plant adaptation, fruit quality, and our health [36].

It has recently been reported that during strawberry fruit ripening, the SPX domain of FaVPT1 perceives InsP_6_ and triggers signaling transduction, then promotes the accumulation of both Pi and sucrose [2]. Indeed, AtVPT1, FaVPT1, and VvVPT1 are confirmed to be low-affinity Pi transporters [4] (Fig. 4). In the present study, FaVPT1-mediated direct transport of sucrose is also confirmed in yeast (Fig. 6). Owing to the accumulation of mainly glucose/fructose in ripening grape berries [37], reasonably, the transport of glucose/fructose in berry vacuoles is now illuminated by VvVPT1 (Fig. 5). Due to almost no sugars in Arabidopsis dry fruit, consistently, no synergetic relationship of Pi with sugar is demonstrated by AtVPT1 (Figs 5 and 6). These findings provide a clue that the diversity of the VPT1 function in sugar transport is likely connected with the type of fruits.

Fruits are the most important sink for sugar storage, which is essential to plant living and metagenesis, thus fruit sugar transport and metabolism are tightly controlled in response to developmental and environmental cues. Notably, *O. sativa* vacuolar Pi efflux transporters (OsVPE1/2) evolved from an ancient plasma membrane glycerol-3-phosphate transporter protein, and are recruited to the vacuolar membrane to catalyse Pi efflux in response to patchy terrestrial environment as an adaptation for land plant living [5]. Given that phosphorus and sugar are essential to plant growth and adaptation, the VPT1 proteins with the SPX sensor and MFS transport domain alternatively transport phosphate and sugar in response to fruit types and soluble sugar status.

Model plant Arabidopsis contains a total of 53 MSTs and seven disaccharide transporters (DSTs), which share little homology at the amino acid sequence, but all have the MFS conserved domain with 12 TMDs [9]. Similarly, the amino acid sequences of VPT1s and sugar transporters have been reported to be quite different, but they have similar three-dimensional structures. On the other hand, some transporters such as MdSTP13a can takes up both hexose and Suc for pollen tube growth in apple [24]. It remains to be determined how each transporter differentiates its molecular cargo. Some conserved residues from TMDs 1, 4, 7, and 10, positioned in the center of MFS transporters [38], potentially contribute to substrate coordination and co-transport coupling.

**Figure 9:**
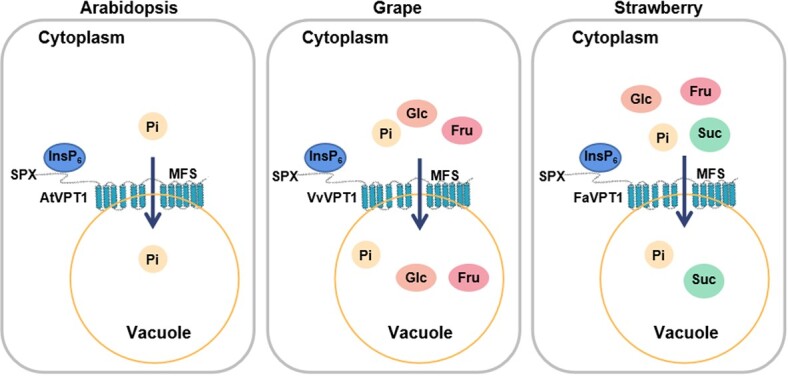
The regulation mechanism diagram of VPT1 proteins mediated transport of soluble sugars and phosphate differs among fruit types. The VPT1 proteins contain conserved SPX structure at the N terminus, which can bind InsP_6_ and triggers signaling transduction. Due to almost no soluble sugars in Arabidopsis dry fruit, AtVPT1 is demonstrated to transport Pi but not soluble sugar into vacuole. Owing to less accumulation of sucrose in ripening grape berries, reasonably, a synergetic relationship of Pi with glucose/fructose in berry vacuoles is illuminated by VvVPT1. During strawberry fruit ripening, FaVPT1-mediated transport of both Pi with sucrose in vacuoles is confirmed in yeast, which may facilitate sucrose, glucose, and fructose accumulation and fruit ripening. Altogether, the VPT1-mediated transport of Pi or sugar varies with soluble sugar component and content in fruit vacuoles.

In conclusion, the VPT1 proteins of fleshy fruits (grape and strawberry) rather than dried fruits (Arabidopsis) have sugar transport activity, thus promoting sugar accumulation and ripening in vacuoles (Fig. 9). In the future, this notion is to be confirmed by more fruits. It is our perspective that manipulation of VPT1 proteins might represent a strategy to improve fruit yield and quality by the synergistic and alterative transport of Pi and sugars.

## Materials and methods

### Plant materials

Tissue samples (roots, stems, leaves, fruits, and buds) were collected from field-grown grapevines. Fruits were sampled at seven stages during the growing season, including expanding (EL_31 to EL_34), véraison (EL_35), and ripening (EL_36 to EL_37) as reported [39]. *FaVPT1* (XM_004298685.2), *VvVPT1* (VIT_202s0025g04540), and *AtVPT1* (AT1G63010) were cloned from strawberry (*Fragaria* × *ananassa* Duchesne) ‘Sweet Charlie’, grape (*V. vinifera*) ‘Kyoho’, and *A. thaliana*, respectively. Tobacco (*N. benthamiana*) for subcellular localization assay were grown in a tissue culture chamber at 25°C under long day conditions (16 h light/8 h dark cycle).

### RNA isolation and real-time quantitative PCR

Total RNA was separately isolated using a Quick RNA isolation Kit (Waryong Biological Technology, Beijing, China) in accordance with the manufacturer’s protocols. RNA was reverse transcribed and qRT-PCR amplification was accomplished according to the manufacturer’s protocols.

*VvEF1-α* was used as a reference gene [40]. The transcription level of genes associated with grape ripening were assessed, including *VvXET1*, *VvCell*, *VvEXPA14*, *VvEXPA19*, *VvSS3*, *VvSUC12*, *VvSUC27*, *VvSPS1*, *VvHXK1*, and *VvHXK2* [41–45]. The primers used for qRT-PCR are shown in Table S1, see online supplementary material.

### Bioinformatics analyses

The similarity analyses of nucleotide and protein sequences were carried out by BLAST (http://blast.ncbi.nlm.nih.gov/Blast.cgi). Conserved domain, transmembrane domains, and 3D structures were predicted in CCD (https://www.ncbi.nlm.nih.gov/cdd), TMHMM (www.cbs.dtu.dk/services/TMHMM/), Phyre2 (http://www.sbg.bio.ic.ac.uk/phyre2/), and Raptorx (http://raptorx.uchicago.edu/).

### Vacuole isolation and subcellular localization

*N. benthamiana* was infected by *Agrobacterium tumefaciens* strain GV3101 harboring Super1300:VvVPT1-GFP and vacuolar marker γ-TIP-mCherry. The vacuole isolation assay of *N. benthamiana* was performed following the method described previously [2]. The fluorescence of VvVPT1 and γ-TIP was observed by confocal laser scanning microscope (Leica, Wetzlar, Germany) with the excitation wavelengths of 488 nm and 561 nm, respectively. These experiments were repeated at least three times.

### Transfection of grape berries by *Agroinfiltration*

The *Agrobacterium* suspension of Super1300:VvVPT1 OE and VvVPT1_326_-pK7GWIWG2-RNAi were prepared according to the method described previously [2]. Thirty synchronised berries were infected for each treatment. In order to avoid the fruit asynchrony problem [46], we selected the adjacent fruits in the same clusters for genetic transformation. Due to the large turgor pressure of the grape berries, we used a modified method for transient transformation of grape berries [15]. Fruits during EL_34 stage were selected, and a syringe needle (0.45 mm diameter) was used to pierce about 30 holes covering about one-third of the grape epidermis. Then, 200 μL *Agrobacterium* suspension was injected into the flesh. The injected grape clusters were encased in fruit bags for 1d, and then cultured under normal conditions. The experiment was repeated at least three times.

### Functional identification of VvVPT1, FaVPT1, and AtVPT1 in the eyeast system

To verify the Pi transport activity of VvVPT1, FaVPT1, and AtVPT1, their CDS was respectively introduced into the yeast expression vector pRS426. Subsequently, the constructs were transferred into the yeast mutant strain YP101 [16, 47, 48]. The experiment was performed as previous reported [2].

The CDS of *VvVPT1*, *FaVPT1*, and *AtVPT1* was respectively introduced into the yeast expression vector pDR195 to verify the sugar transport activity. The construct was transformed into the hexose transport-deficient yeast strain EBY.VW4000 or Suc transport mutant strain SUSY7/ura3 [20], respectively. Yeast strains were harvested and adjusted to an OD_600_ of 0.2, and then spotted in serial dilutions and cultured on the medium, which was supplemented with 2% (w/v) Suc, Mal, Glc, or Fru as the sole carbon source. Yeast cultures were incubated 3 d at 30°C before photography.

To explore whether the MFS domain in SPX-MFS can perform its transport function alone, the three sequences of truncated SPX domain (ATG + MFS domain) were respectively constructed and transformed into the YP101, EBY.VW4000, and SUSY7/ura3, respectively to determine the influence of the SPX domain on the transport activity.

### Glc uptake assay using fluorescent 2-NBDG in the yeast system

The 2-NBDG uptake assay was mainly performed as described previously [19]. Briefly, the yeast cells of WT, EBY.VW4000, and positive transformants were initially cultured in Glc free YP liquid until OD_600_ = 1.2–1.5. Then the cells were harvested by centrifugation, and resuspended in the aforementioned YP liquid with 60 μM of 2-NBDG at 30°C for 3 h. Finally, the cells were washed three times with PBS (pH = 7.4) and visualized with a confocal laser scanning microscope (Leica SP8 lightning confocal microscopy) under 40$\times$ objective lens using GFP filter with the excitation wavelengths of 488 nm.

### Suc transporter assay using fluorescent esculin substrate

Yeast cells of WT, SUSY7/ura3, and positive transformants were cultured in YP liquid to mid log phase. After being collected by centrifugation, the cells were resuspended in phosphate buffer (25 mM Na_2_HPO_4_, pH = 4) with 1 mM esculin. Subsequently, the cells were cultured in a shaker at 30°C for 1 h, and then washed three times with PBS (pH = 7.4). Finally, the cells were observed by a confocal laser scanning microscope (Leica SP8 lightning confocal microscopy) using excitation wavelengths of 420–460 nm.

### Yeast growth curve

Yeast growth curve analysis was described previously [49]. To test the transport affinity to Suc of FaVPT1, yeast cells of WT, FaVPT1/SUSY7/ura3, and AtVPT1/SUSY7/ura3 were cultured in YP liquid with different concentrations of Suc. Similarly, yeast cells of WT, VvVPT1/ EBY.VW4000, and AtVPT1/EBY.VW4000 were treated with different concentrations of Glc. All the treatments were adjusted to an initial OD_600_ of 0.2, then yeast growth was determined at 600 nm after 48 h at 30°C in a shaker.

### Subcellular localization in yeast

The CDS of *VvVPT1*, *FaVPT1*, and *AtVPT1* was respectively introduced into the yeast expression vector pDRF-GFP to verify the subcellular localization as described previously [50]. The construct was transformed into the WT yeast strain. The plates were incubated at 30°C for 3 d and positive clones were confirmed and sequenced. The yeast cells were resuspended in sterile 0.9% NaCl solution. Fluorescence of VPT1-GFP was analysed using a Leica confocal microscope at wavelengths of 488 and 561 nm for excitation and emission, respectively.

### Incubation of flesh discs *in vitro*

The experiment was performed as described previously [51]. To investigate whether the expression of *VvVPT1* is regulated by sugar, fruits at EL_34 stage were selected for incubation assays. After the skin and seeds were removed, the flesh was cut into 1 mm discs, and then equally distributed to 250 mL flasks containing incubate liquid with different concentrations of Suc, Glc, and Fru for 0.5 h, 1 h, 2 h, 3 h, and 4 h. The discs incubated for 30 min were used as control (each treatment including five fruits per replication). Processed discs were washed with ddH_2_O and immediately frozen in liquid nitrogen and kept at −80°C until use. The experiment was performed with three replications.

### Statistical analysis

Statistical analysis was performed using SigmaPlot version 12.0 (Systat Software). Data are reported as the mean ± SE. Different lowercase letters above the bars (such as a, b, c, d, e, f, g, and h) indicate a significant difference from each other according to Duncan’s multiple range test (*P* < 0.05). Asterisks indicate significant differences between each treatment, assessed by Student’s t-test.

## Acknowledgments

We thank Prof. Zhenxian Zhang, Lailiang Cheng and Dong Meng for providing the yeast stain SUSY7/ura3; Xuexian Li for providing the yeast stain EBY.VW4000; Yifang Chen and Erin K. O’Shea for providing the yeast stain YP101. This work was supported by the National Natural Science Foundation of China (Projects 32030100; 32102362), Natural Science Foundation of Beijing (6222004), National Key Research and Development Program (2018YFD1000200), Science and Technology Innovation Support Program (BUA-HHXD2022005), and Research and Innovation Ability Improvement Program for Young Teachers of Beijing University of Agriculture.

## Author contributions

Y.H. and Y.S. conceived the study and managed the projects; Q.B., X.C., and Z.Z. performed the experiments. Q.B., X.C., Z.Z., and Y.H. performed data analysis. Q.B., X.C., Z.Z., Y.H., and Y.S. wrote and revised the article.

## Data availability

The data underlying this article are available in the article and in its online supplementary material.

## Conflict of interests

The authors declare that no competing interests exist.

## Supplementary data

Supplementary data is available at *Horticulture Research* online.

## Supplementary Material

Web_Material_uhac260
