# Supplementary material for: Vacuolar Phosphate Transporter1 (VPT1) may transport sugar in response to soluble sugar status of grape fruits
Source: Hortic Res. 2022 Nov 22;10(2):uhac260. doi: 10.1093/hr/uhac260 (PMC10392026; doi:10.1093/hr/uhac260)
Supplement: Web_Material_uhac260 [file web_material_uhac260.docx]

# Supplemental files

## Supplemental Fig. 1. Predicted structures of FaVPT1, VvVPT1, and AtVPT1.

(a) Amino acid sequences comparison and conserved domain analysis of FaVPT1, VvVPT1, and AtVPT1. TM: transmembrane domain.

(b) Predicted schematic diagram for FaVPT1, VvVPT1, and AtVPT1.





##

## Supplemental Fig. 2. Expression levels of *VvVPT1* in fruit treated with different sugars.

(a) qPCR analysis of *VvVPT1* expression in response to different concentration of Glc.

(b) *VvVPT1* expression in response to different concentration of Fru. Data are shown as the mean ± SE (n= 3).

**

**

## Supplemental Fig. 3. The sugar response related *cis* element analysis of upstream sequences of the ATG initiation codon of *VvVPT1*.

The black line represents upstream sequences of the ATG initiation codon of *VvVPT1*. The red vertical lines represent sugar response elements (SUREs) and SURE-Like elements. The green vertical lines represent W-box elements. The brown vertical lines represent G-box elements.

**

**

## Supplemental Fig. 4. Predicted three-dimensional structure for VvVPT1, FaVPT1, and AtVPT1.

Three-dimensional structures of VvVPT1 (top panel), FaVPT1 (middle panel), and AtVPT1 (bottom panel) were predicted using Phyre2 (http://www.sbg.bio.ic.ac.uk/phyre2). The front, top, and bottom views of the predicted protein structures are shown in each panel. N: N terminal; C: C terminal.





## Supplemental Fig. 5. Subcellular localization of FaVPT1, VvVPT1, and AtVPT1 in yeast.

The panels, from left to right, show the GFP signal (green), the bright field (BF), an overlay image from the same yeast cell. Bars: 2 μm.





## Supplemental Table 1: Primer sequences used in this study.

| Names of primer pair or genes | Forward primer (5' to 3') | Reverse primer (5' to 3') | Notes |
| --- | --- | --- | --- |
| VvVPT1-426 | ttcctgcagcccgggggatcc atggttgcctttgggaaaaagctg | ctccaccgcggtggcggccgc ttaatagagagagttgtaggtaaag | Construct *VvVPT1* into pRS426 vector |
| FaVPT1-426 | ttcctgcagcccgggggatcc atggtggcctttgggaaa | ctccaccgcggtggcggccgc tcaatagagagagttgtaggtatagc | Construct *FaVPT1* into pRS426 vector |
| AtVPT1-426 | ttcctgcagcccgggggatcc atggtggcttttgggaaa | ctccaccgcggtggcggccgc tcaatagagtgagttataagtacaac | Construct *AtVPT1* into pRS426 vector |
| VvVPT1-195 | cctcgacccagcctcgag atggttgcctttgggaaaaagctg | aagtccaaagctggatcc ttaatagagagagttgtaggtaaag | Construct *VvVPT1* into pDR195 vector |
| FaVPT1-195 | cctcgacccagcctcgag atggtggcctttgggaaa | aagtccaaagctggatcc tcaatagagagagttgtaggtatagc | Construct *FaVPT1* into pDR195 vector |
| AtVPT1-195 | cctcgacccagcctcgag atggtggcttttgggaaa | aagtccaaagctggatcc tcaatagagtgagttataagtacaac | Construct *AtVPT1* into pDR195 vector |
| *VvVPT1* OE | ctgcaggggcccggggtcgac atggttgcctttgggaaaaagctg | catggtaccggatccactagt atagagagagttgtaggtaaag | Super1300:VvVPT1-GFP |
| *VvVPT1* RNAi | gggg acaagtttgtacaaaaaagcaggcttc atcaacctgcccttcctctc | gggg accactttgtacaagaaagctgggtc gaacacttgagcaacagcca | VvVPT1_326_-pK7GWIWG2-RNAi |
| VvVPT1_155−698_-426 | ttcctgcagcccgggggatcc atgttgcagcaagtgttcaagc | ctccaccgcggtggcggccgc ttaatagagagagttgtaggtaaag | Construct *VvVPT1_463−2097_* into pRS426 vector |
| FaVPT1_147−696_-426 | ttcctgcagcccgggggatcc atgcgtgctaatcatccttac | ctccaccgcggtggcggccgc tcaatagagagagttgtaggtatagc | Construct *FaVPT1_439−2091_* into pRS426 vector |
| AtVPT1_162−708_-426 | ttcctgcagcccgggggatcc atgtctcagcttcaacaagttt | ctccaccgcggtggcggccgc tcaatagagtgagttataagtacaac | Construct *AtVPT1_484−2127_* into pRS426 vector |
| VvVPT1_155−698_-195 | cctcgacccagcctcgag atgttgcagcaagtgttcaagc | aagtccaaagctggatcc ttaatagagagagttgtaggtaaag | Construct *VvVPT1_463−2097_* into pDR195 vector |
| FaVPT1_147−696_-195 | cctcgacccagcctcgag atgcgtgctaatcatccttact | aagtccaaagctggatcc tcaatagagagagttgtaggtatagc | Construct *FaVPT1_439−2091_* into pDR195 vector |
| AtVPT1_162−708_-195 | cctcgacccagcctcgag atgtctcagcttcaacaagttt | aagtccaaagctggatcc tcaatagagtgagttataagtacaac | Construct *AtVPT1_484−2127_* into pDR195 vector |
| VvVPT1-qPCR | ttttggcccaacatgcactt | gaacacttgagcaacagcca | qRT-PCR |
| VvSUC12 | ctgaagcaggctgaggttga | cggcggcaatcatacaactg | qRT-PCR |
| VvSUC27 | ctcttctcctcccactccca | aactaccggctgcacaatca | qRT-PCR |
| VvHXK1 | cgcggttggacaagatgtag | tacccaagatcactgcagca | qRT-PCR |
| VvHXK2 | gtcgtggaggtttgtgacac | atctccaaccccagaagctc | qRT-PCR |
| VvSPS1 | gagcgaaaactacgagccag | ataggcttgcgaggattggt | qRT-PCR |
| VvSS3 | gccctgcatggttcaattga | gtcaagccttgccatggaaa | qRT-PCR |
| VvEXPA14 | caagttaggagggcagaagtttgt | caacgatgaccccaaatggt | qRT-PCR |
| VvEXPA19 | aaatgggcacaaggagttgatag | ctcaaatggttggcaatgga | qRT-PCR |
| VXET1 | acaacgaagactaccctggg | accttcttacggggacatcg | qRT-PCR |
| VvCEll | tggatccgcagaatggatgt | ccatcatggctcaaagccttag | qRT-PCR |
| VvEF1-α | gaacgttgctgtgaaggatctc | cgcctgtcaaccttggtcatga | qRT-PCR |
